# Correlation between Implant Geometry, Bone Density, and the Insertion Torque/Depth Integral: A Study on Bovine Ribs

**DOI:** 10.3390/dj7010025

**Published:** 2019-03-05

**Authors:** Danilo Alessio Di Stefano, Paolo Arosio, Vittoria Perrotti, Giovanna Iezzi, Antonio Scarano, Adriano Piattelli

**Affiliations:** 1 Dental School, Vita-Salute University IRCCS San Raffaele, 20132 Milan, Italy; 2 Private Practitioner, Vimercate, 20871 Monza-Brianza, Italy; p.arosio@libero.it; 3 Department of Medical, Oral and Biotechnological Sciences, University of Chieti-Pescara, 66100 Chieti, Italy; vittoria.perrotti@unich.it (V.P.); gio.iezzi@unich.it (G.I.); apiattelli@unich.it (A.P.); 4 Department of Medical, Oral and Biotechnological Sciences and CeSi-MeT, University of Chieti-Pescara, 66100 Chieti, Italy; antonio.scarano@unich.it

**Keywords:** primary stability, bone density, implant morphology, torque–depth curve integral, insertion torque, removal torque

## Abstract

During insertion of dental implants, measurement of dynamic parameters such as the torque-depth curve integral or insertion energy might convey more information about primary stability than traditional static parameters such as the insertion or removal torque. However, the relationship between these dynamic parameters, bone density, and implant geometry is not well understood. The aim of this investigation was to compare static and dynamic implant stability measurements concerning three different implant designs when implants were inserted into bovine bone ribs and dynamic parameters were collected using an instantaneous torque measuring implant motor. Standard implant osteotomies were created in segments of bovine ribs. After measuring the bone density using the implant motor, 10 cylindrical, 10 hybrid tapered-cylindrical, and 10 modified cylindrical implants were placed, and their primary stability was assessed by measuring the torque–depth curve integral, along with insertion and removal torque. The relationship between these quantities, bone density, and implant geometry was investigated by means of regression and covariance analysis. The regression lines describing the relationship between the torque–depth integral and bone density differed significantly from those describing the relationship between insertion torque, removal torque, and bone density for all three designs. The torque–depth curve integral provides different information about immediate primary stability than insertion and removal torque and in certain clinical conditions might be more reliable than these static parameters for assessing implant primary stability. Further research should be carried out to investigate the findings of the present study.

## 1. Introduction

The absence of movement immediately after implant insertion is one of the most important factors affecting implant osseointegration [1,2]. Primary stability depends on a number of variables, including the bone quality and density at the placement site [3,4], the site-preparation protocol [5,6,7], and the implant geometry, both micro- and macroscopic [4,8,9,10]. Taking into account the bone density and quality at any given implant-placement site, the surgeon should choose the implant size and geometry that best fits the site. The implant site-preparation protocol should be accurately chosen to reduce implant micro-movement below the 50–150 µm threshold and provide proper stability and prosthetic support in accordance with the planned loading protocol (delayed, early, or immediate) [2,11,12,13,14]. Crucial steps of this workflow include the measurements of bone density and implant primary stability.

Reliable in vivo bone density measurements are not always feasible. While computed tomographic (CT) bone density measurements are trustworthy, concerns about patient radiation exposure have arisen [15]. Cone-beam CT (CBCT) technology enables reduction of the amount of radiation delivered [16]; however, doubts still exist about the reproducibility of the results, as calibration of CBCT machines is usually brand-dependent and not known to the user [17,18,19]. 

In clinical practice, most surgeons still rely on subjective sensations during drilling, scoring bone density according to the classifications of Lekholm and Zarb [20], Misch [21], and/or Trisi and Rao [22]. Primary stability may be measured in the clinical setting by assessing insertion torque (IT) or the implant stability quotient (ISQ) by means of resonance frequency analysis (RFA) [23]. It has been shown that IT and RFA convey different information about the bone–implant interaction [24,25]. To overcome this ambiguity, some authors have proposed to assess primary stability by measuring insertion energy (IE), that is, the total energy needed to place the implant into its site [26,27,28]. Preliminary findings have shown that IE may be more reliable than RFA or IT at identifying those situations where sufficient primary stability can be achieved even in softer bone [26] and more reproducible at quantifying the primary stability enhancement provided by underpreparation [29]. At present, though, the relation between IE, RFA, and IT is far from clear [28].

Concerning implant geometry, the original endosseous implants introduced by Branemark were cylindrical. Later, tapered implants were introduced to improve esthetics, facilitate implant placement between adjacent natural teeth, and improve the outcomes of immediate implant placement after tooth extraction [30]. The tapered design allows the gradual expansion of the bone ridge, therefore minimizing the stress to the surrounding bone [8]. Tapered implants have been shown to be more stable in low density sites [31,32], but some studies have also found a lower success rate for them than that for cylindrical implants [33] and greater marginal bone loss after one year of functioning [34]. In vitro biomechanical studies comparing tapered and cylindrical implants have provided conflicting results [14,35,36], and the biomechanical behavior of tapered implants is still not completely understood [37]. 

Recently, a torque-measuring micromotor has been introduced that enables quantitative intra-operative and site-specific bone density measurement during implant-site preparation. The micromotor allows measuring bone density at an intermediate step of implant site preparation by means of a dedicated probe; such density measurement is based on the principle that cutting resistance at threading is a good estimator of bone quality at the placement site [38], as shown by Friberg et al. in studies on pig ribs and jaw autopsy specimens [39,40]. Bench tests on polyurethane foam blocks mimicking cancellous bone showed that the average torque measurements provided by the system correlated significantly with the actual block density and enabled measurement of the average statistical error introduced by the device–operator system during bone density assessment [41]. The same tests made it possible to build calibration curves for the device both with and without irrigation. These torque measurements were also shown to correlate significantly with histomorphometric bone density measurements of bovine bone ribs [41]. When used in human subjects, the torque-measuring micromotor was shown to provide operator-independent bone density measurements and correctly discriminate between the anterior and posterior areas of both arches [41,42]. 

During implant insertion, the micromotor calculates a dynamic, primary-stability-measuring parameter, the integral (I) of the torque–depth curve. The I parameter has been shown to correlate significantly with the bone-to-implant contact (BIC) both in a study on bovine ribs and in the clinical setting [43,44]. In bench tests on polyurethane foam blocks I has been shown to measure implant primary stability reliably and with a greater sensitivity to bone density variations than IT, reverse torque (RT), and ISQ [45]. 

While the relation between IT, RT, bone density, and implant geometry has been extensively studied [24,31,32], no investigations have ever been carried out concerning how implant geometry may affect I at different bone densities. The present study aimed therefore to investigate the relation between implant geometry, bone density, and I by analyzing data collected while using the micromotor to place implants with three different designs in bovine ribs. Another goal was to investigate whether I, IT, and RT convey different information about implant primary stability in relation to bone density and implant macrogeometry.

## 2. Materials and Methods 

In this ex vivo study nine segments of bovine ribs were used after removing the periosteum. All surgical procedures were carried out by experienced operators. 

**Figure 1:**
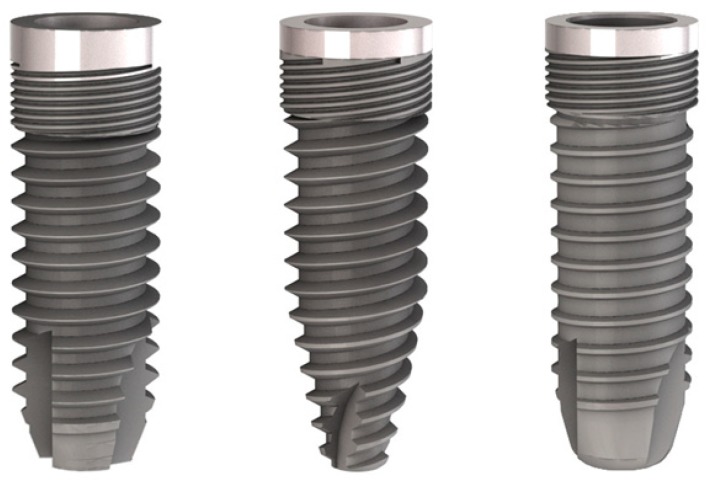
The three implants used in the present study. Their geometrical features are provided in Table 1. Left, the cylindrical Stone implant; center, the cylindrical-tapered Tiger Due implant; right, the modified cylindrical Aries Due implant.

A total of 30 osteotomies were created in the bovine bone samples to accommodate three different types of implants (IDI Evolution, Concorezzo, Italy) (10 osteotomies for each implant type): cylindrical (Stone), cylindrical-tapered (Tiger Due), and cylindrical-modified (Aries Due). Osteotomies were carried out consecutively, placing all cylindrical implants first, followed by all cylindrical-tapered, and then all cylindrical modified ones. Osteotomies were carried out to leave enough room between two adjacent implants (about 1–1.5 cm), and a different segment was used when no more room was available on the one being used. All implants shared the same double-etched, sandblasted surface (Figure 1 and Table 1). 

The computerized implant micromotor (TMM2, IDI Evolution) was used both for intra-operative analysis of bone density and for primary stability measurement at the time of implant placement. The micromotor provides, both when measuring bone density and when assessing primary stability, the set of values shown in Table 2. 

**Figure 2:**
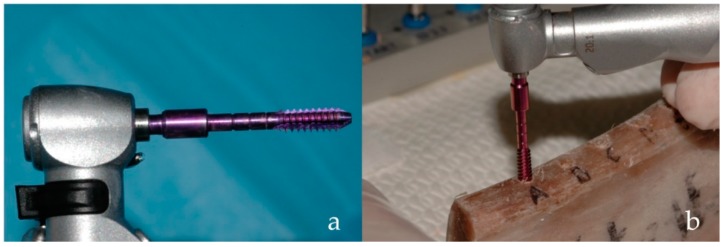
The probe used to measure the density of the bone specimen. The probe has an inverse cone shape (**a**), enabling measurement the friction encountered by the first thread only during its descent (**b**). The average of all instantaneous torque values measured throughout the probe’s descent, Cm, has been shown to be a reliable measurement of bone density [46].

Bone density measurements were carried out using a special 2-mm-wide reading drill, featuring 3-mm-wide equally spaced threads shaped as a 1-degree reverse cone (patented, Figure 2a). 

Insertion depth and direction of the perforations were defined by using a 2.2-mm-diameter pilot drill to perforate the cortical bone. Each site was then prepared to the desired depth following the drilling sequences recommended by the manufacturer. The cortex of the upper portion of the bone specimens was then removed using a 3.0 mm reamer drill.

**Figure 3:**
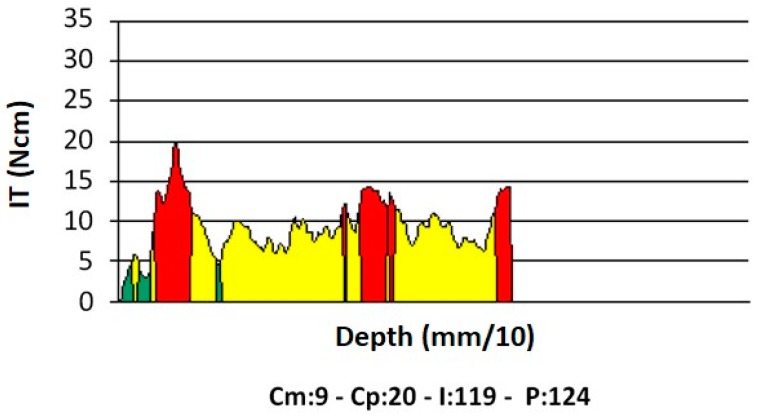
An example of a torque–depth plot generated from the micromotor measurements of bone density and during implant insertion. Cm, average torque; Cp, peak torque; I, integral of the torque–depth curve; P, depth reached by the probe/implant in tenths of a millimeter.

The implant motor was switched into its “read mode,” and the special reading drill, rotating at a preset speed (35 rpm), was used to evaluate the density [46,47] of each bone site up to the pre-drilled depth (Figure 2b). Such speed, equal to that also used to place implants, is factory-set and was used to build all calibration curves of the device; different speeds may produce different results. During measurement, the device displayed a torque/depth graph showing how the instantaneous torque varied according to the probe depth, together with the average torque (Cm), peak torque (Cp), and I (the torque–depth curve integral) (Figure 3).

Bone densities were then calculated from the Cm values using the formula provided by Di Stefano et al. [46], for density measurement with no irrigation (bone density D (g/cm^3^) = (Cm + 11.93)/43.97). 

Implant placement followed using the same micromotor at a constant preset speed of 35 rpm. Implant sites for cylindrical implants (diameter 4.0 mm), were prepared using a 3 mm twist drill followed by a 4 mm reamer drill. For cylindrical-tapered implants (diameter 4.3 mm), sites were prepared using a 3 mm twist drill, followed by a 4.3 mm reamer drill. Finally, for cylindrical-modified implants (diameter 4.0 mm), sites were prepared using a 3 mm drill, followed by a 4 mm reamer drill. During implant placement, the device again displayed the torque/depth graph and the same three parameters—Cm, Cp, and I (Table 2). The peak torque Cp is commonly known as the insertion torque (IT). It should be noted that, under the conditions of the present study (a constant implant insertion speed and evenly spaced implant threads), I is equal to the insertion energy (IE) multiplied by a constant factor. 

All measurements at probing and at insertion were stored in the device’s solid-state memory and were later downloaded to a personal computer for statistical analysis. 

For each implant placed, the RT also was measured as follows. One experimenter (P.A.) connected a high precision manual dynamometer (ATG6CN Torque Gauge, Tohnichi Mfg. Co., Tokyo, Japan) to the implant and manually applied an increasing counter-clock torque up to the initial unscrewing of the implant. The needle on the dynamometer display corresponded to the torque value measured. An independent experimenter read the display.

### Data Analysis

To investigate the relationship between bone density and primary stability, I–density, IT–density, and RT–density plots were drawn, and linear regression analyses were performed. To investigate the effect of the implant geometry on primary stability, average values for each implant type and each primary stability parameter were calculated and compared by means of ANOVA tests followed by post-hoc Bonferroni tests. To investigate whether I, IT, and RT convey similar or different information, slopes of I–density, IT–density, and RT–density lines corresponding to the same implant type were compared using ANCOVA analysis [48]. All values in this work are provided as mean ± standard deviation (SD). Statistical calculations were performed using standard statistical software (Origin 9.0, OriginLab, Northampton, MA, USA). The methodology was reviewed by an independent statistician. 

## 3. Results

**Figure 4:**
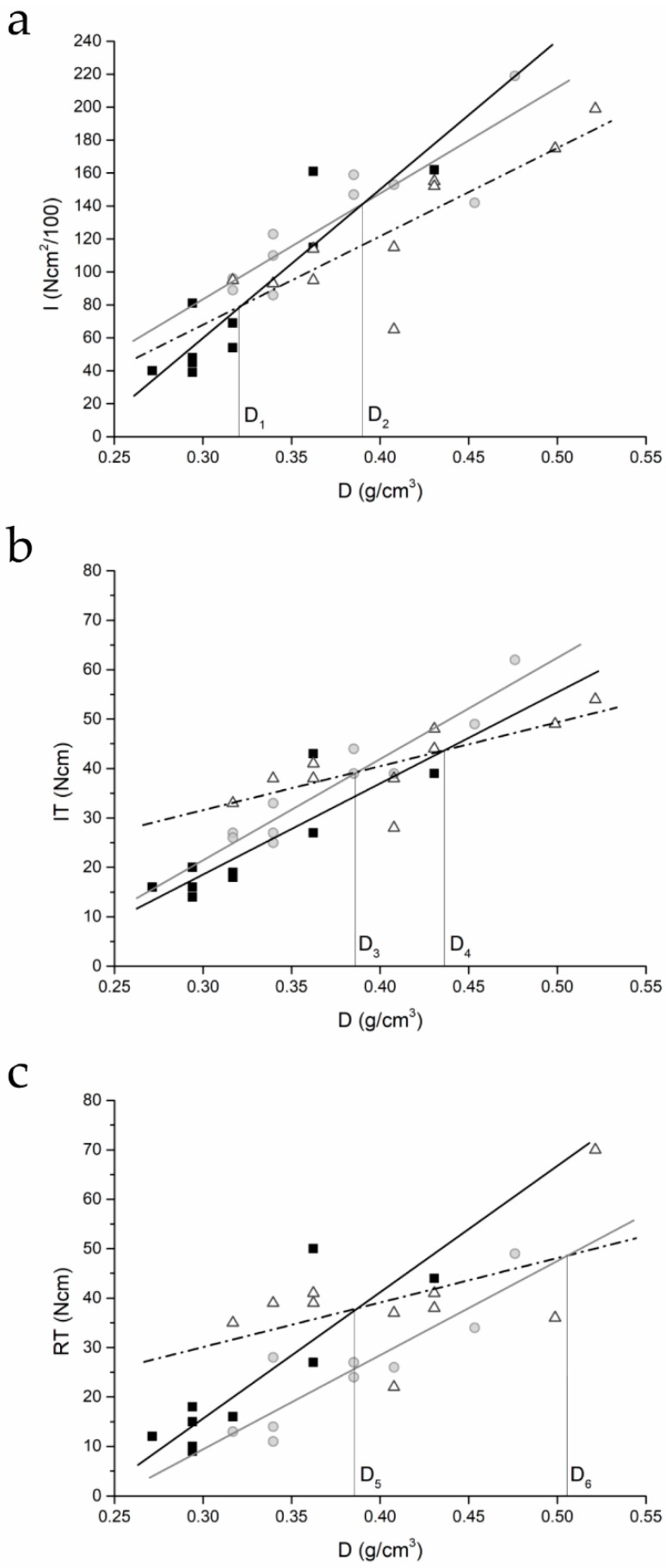
(**a**) Integral of the torque–depth curve (I)–density, (**b**) insertion torque (IT)–density, and (**c**) reverse torque (RT)–density plots for the three implants (square, cylindrical; circle, tapered; triangle, cylindrical-modified) placed after standard site preparation. The plots also show the density thresholds (D_x_) where the lines cross.

Results of the regression analyses are provided in Figure 4 and Table 3. 

**Figure 5:**
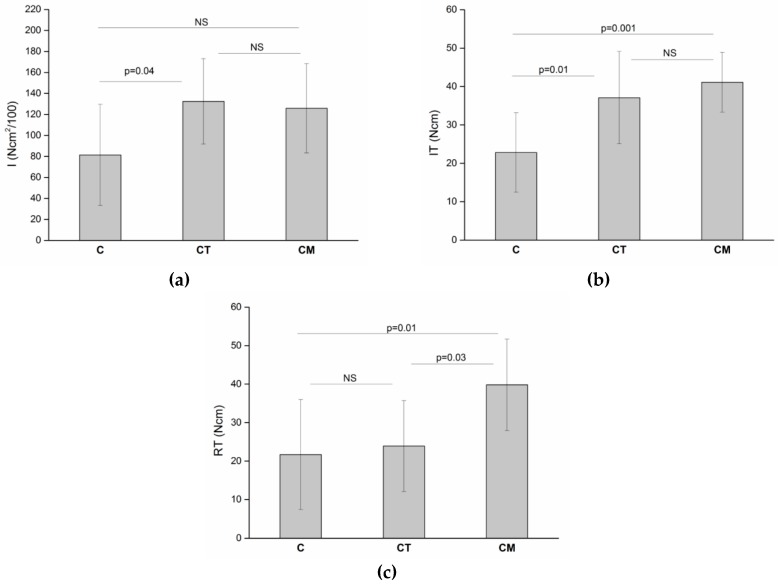
(**a**) Average I, (**b**) IT, and (**c**) RT values for the three types of implants (C, cylindrical; CT, cylindrical-tapered, CM, cylindrical-modified) and significance of the post-hoc Bonferroni paired comparison.

The I–density plot shows two bone density thresholds, D1 and D2, below which the I for cylindrical-tapered and cylindrical-modified implants is greater than that of cylindrical implants. This does not hold for IT–density plots. The IT–density line for the cylindrical-tapered implants is always higher than that for cylindrical implants and two density values exist, D3 and D4, below which IT is greater for modified cylindrical implants than for cylindrical or tapered ones. RT–density plots show that, unlike the IT–density results, the RT–density line for tapered implants is always lower than that for cylindrical implants. Two density values, D5 and D6, exist below which the RT needed to remove this type of implant is greater than that necessary for the removal of the other two types. Average I, IT, and RT values are provided in Table 4 and Figure 5.

The ANOVA testing found significant differences among the three implant types (*p* < 0.05 in all cases, Table 4). Post-hoc Bonferroni tests (Figure 5) showed that differences between one type of implant and another one were not equally significant concerning all three parameters. The average I for cylindrical implants was significantly different from that of cylindrical-tapered implants (*p* = 0.04). So was the average IT (*p* = 0.01), but the difference was not significant when RT was assessed. Average I of cylindrical implants was found to be not significantly different from that of cylindrical-modified ones, while average IT and average RT did differ significantly (*p* = 0.001 and *p* = 0.01, respectively). Average RT was found to be significantly different between cylindrical-tapered and cylindrical-modified implants (*p* = 0.03), but that was not observed for average I and average RT. Finally, results of ANCOVA tests (Table 5) showed that the slopes of the I–density regression lines were significantly different from those of the RT–density regression lines for all three types of implants. When the slopes of the IT–density regression lines were compared to those of the RT–density ones, no significant difference could be observed for any implant type.

## 4. Discussion

The results of the present study indicate that the relationship between bone density and primary stability of implants with various shapes may vary significantly, depending upon which parameter is chosen to assess the primary stability. Remarkably, when implants were placed according to the manufacturer’s standard protocol, the primary stability of the cylindrical-tapered implants, when assessed using I, was shown to be greater than that of cylindrical implants for bone density values below a certain threshold, while cylindrical implants showed a greater primary stability for greater bone density values. This behavior was not observed when implant stability was measured either using IT or RT. Then the cylindrical-tapered implants always displayed greater primary stability than cylindrical ones.

Results concerning IT and RT measurements are consistent with some but not all biomechanical studies that have examined the relationship between primary stability and bone density when tapered and cylindrical implants have been placed in pig or bovine ribs or polyurethane bone blocks [8,35,36]. Valente et al. [36] found that tapered implants placed in polyurethane bone blocks had greater primary stability, measured through IT, in the density range between 0.24 and 0.64 g/cm^3^. Unfortunately, they did not measure the density of the pork ribs used in the study, so their data cannot be compared to those of the present study. Toyoshima et al. [35] compared IT for tapered and cylindrical implants placed in porcine ribs and iliac crests but did not find any significant differences between the two types of implants. These researchers also did not provide any measure of density for their bone samples. Dos Santos et al. [8] found that tapered implants required a greater IT than cylindrical ones when both were placed in high-molecular-weight polyethylene mimicking cortical bone.

The present study shows that, on average, cylindrical-tapered implants require a greater IT for their insertion. They also required more IE, as measured by the I parameter. Yet these findings may be regarded as true only when considering an averaged value across a relatively wide density range; differences exist if a more detailed analysis that considers the relationship linking different primary stability measures with bone density is carried out. The difference between the I–density, IT–density, and RT–density plots, the first showing the I–density regression lines intersecting while the other two not doing so, merits discussion. This finding is consistent with observations by Wang et al. [49], who focused on tapered implants and found a lack of correlation between the IE and the final insertion torque (in most cases equal to IT). The authors called for further research on this question. 

Previous studies by the authors’ research group have shown that the I parameter is more sensitive to density variation than IT [45]. Degidi et al. [26] have shown that IE could better detect those situations where sufficient primary stability can be achieved even in softer bone (as classified according to Lekholm and Zarb’s criteria) [20]. IE might also better detect primary stability enhancements provided by underpreparation than would RFA or IT measurements [29]. Park et al. [28] have shown that IE correlates differently with IT during insertion of the apical (lower correlation, Pearson’s r = 0.356), middle (Pearson’s r = 0.513), and coronal part of the implant (higher correlation, Pearson’s r = 0.780). Tapered implants gain their primary stability by exerting controlled compressive forces on the cortical bone layer that increase as the implant is inserted [50]. Accordingly, tapered implants may involve a greater energy exchange at their coronal portion and a smaller one at their apical one than cylindrical fixtures during placement. If one supposes also that density variations throughout the length of the osteotomy may affect the friction encountered by a cylindrical implant more than that encountered by a tapered implant (because of the different shapes), this would yield I–density lines that intersect as in Figure 4. If this were the case, the total energy exchanged by the implants would be the sum of two quantities. The first quantity, which can be expressed as a constant (k), is given by the final interaction with the cortical layer, which is always greater for tapered implants. The second quantity is represented by the energy exchanged at the level of the cancellous bone, which increases less for tapered implants than for cylindrical implants as density increases. If these conditions held, insertion energy values for tapered (IEt) and cylindrical implants (IEc) might then be expressed as IE_t_ = k_t_ + a(d) and IE_c_ = k_c_ + b(d), where k_t_ and k_c_ are constants, with k_t_ > k_c_, and a(d) and b(d) are two (possibly linear) functions of density, a(d) increasing less than b(d) as density increases. This would provide intersecting lines such as those in Figure 4. Under this hypothesis, IT–density lines would still be expected to be greater for tapered implants than cylindrical ones for all density values, as in Figure 4b.

These hypotheses should be examined further. Indeed, the dimensional parameters of the implants used in the present study may have enhanced the differences between the I–density and IT–density curves for cylindrical and cylindrical-tapered implants. Both implants were inserted in a 3-mm-diameter hole, but the head of the tapered implant was larger (4.3 versus 4.0 mm). This may have favored a greater exchange of energy between the coronal portion of tapered implants and the cortical bone layer.

On the basis of the above considerations, it could be assumed that a possible clinical advantage to measuring primary stability using the integral (I) (and, similarly, the IE) instead of IT might exist. The scenario that emerges from results of the present paper is that when the implant is subjected to surrounding topological conditions that imply a high IT value (such as, for example, the presence of a thick cortical layer), the degree of stabilization provided by the other bone portions around the implant, being unknown, might be overestimated by the surgeon. Such overestimation might lead the surgeon to favor early or immediate loading protocols over delayed ones even when the underlying conditions would not be actually favorable, therefore increasing the probability of early implant failure. Results of the present study indicate that this might be avoidable by measuring primary stability using energy-exchange parameters such as I. The first clinical implication is therefore that measuring primary stability using I instead of IT, the surgeon might have more reliable information concerning stability itself and might better plan the following loading protocol (immediate, early, or delayed), thus enhancing the short- and long-term implant and prosthetic survival and success rates. Further, results of the present study show that differences in implant stability between implants having different geometries may be small and negligible when stability is measured by IT and be much greater when measured by I; the cylindrical-tapered implants used in the present study had, in low-density bone, a much greater stability than the cylindrical one, if stability was measured using I, and should be therefore preferred in low-density sites. If stability was measured using IT, the difference was much smaller; in the clinical setting, this might have led the surgeon to regard the two shapes as equivalent, while he/she should have preferred using the cylindrical-tapered over the cylindrical one. The second clinical implication of the present study is, therefore, that using I instead of IT to measure primary stability might allow detection of differences in primary stability between different implant geometries that could not be detected otherwise, again enhancing the probability of carrying out more adequate loading protocols. From a wider perspective, the clinical advantage of using I instead of IT to measure implant primary stability might be allowing better characterization of the implant-specific response to a given site preparation protocol according to its macro- and micro geometrical features, and the features of the bone it will be placed into. This might lead to a brand- and shape-specific fine characterization of the response, in term of primary stability, of the implant to the patient-specific and site-preparation specific features at its insertion. 

Limitations of the present study include the fact that implant insertion was carried out with no irrigation. When implants were inserted in polyurethane foam blocks with or without irrigation [45], irrigation was unexpectedly observed to not affect the I–density curves, which were superimposable on those achieved in dry conditions, within the limits of the experimental errors; this finding, yet, might not be hold true for a different experimental system, as bovine ribs are, and experiments should be repeated also under irrigation. Further, bovine rib segments used in the present study were found to have quite different bone densities; while such differences were not enough to jeopardize the study, as reliable stability–density lines could still be drawn, a better experimental design would be that of not placing implants having the same geometry consecutively in the same segment but to sort them among different bone segments. 

Another limitation of the present study lies in having not correlated the thickness of the cortical bone layer to the implant stability; while all sites were prepared according to the same protocol, the difference observed between the IT, RT, and I–density curves being therefore real, further studies should be carried out (and are actually being carried out by the authors) to investigate this point. 

## 5. Conclusions

In conclusion, when the primary stability of cylindrical and cylindrical-tapered, as well as cylindrical-modified, implants is measured using the dynamic I parameter (a quantity proportional to the energy needed to position the fixture), a linear relation with bone density is observed, one that differs from that shown by the other primary-stability-measuring parameters, namely IT and RT. This finding may indicate that I conveys different information, and in certain clinical conditions could be more reliable than IT and RT in measuring implant primary stability. Based on the results of the present study, the authors recommend measuring primary stability using a dynamic parameter such as I as it might provide additional information to that provided by measuring IT. Results of the present study also show that, limited to the brand under investigation, cylindrical-tapered implants should be preferred to cylindrical, or cylindrical-modified implants, when placement is to be carried out in low bone density sites.

Additional research should be conducted to further investigate these findings, especially in terms of their validity in the clinical setting.

## Figures and Tables

**Table 1:** Geometrical features of the implants used in the present study and corresponding final drilling sequence. C indicates cylindrical, CT cylindrical-tapered, and CM cylindrical-modified. All measures are given in mm.

Implant Characteristics			
Implant type	C	CT	CM
Commercial name	Stone	Tiger Due	Aries Due
Head diameter	4.0	4.3	4.0
Thread diameter	4.0	4.3	3.75
Body diameter	3.2	3.6	3.4
Length	12	12	12
Thread	Single	Double	Single
Thread type	Standard V-thread	Standard V-thread and spiral	Standard V-thread
Thread pitch	0.75	0.75 (lead 1.50)	0.75
Thread depth	0.4	0.4	0.175
Threading at neck	Triple threaded, 0.75 mm pitch	Six-fold threaded, 1.50 mm pitch	Triple threaded, 0.75 mm pitch
Final drilling sequence			
Final drill	3	3	3
Countersink drill	4	4.3	4

**Table 2:** A summary of the measures provided by the micromotor.

Measure	Symbol as Provided by the Micromotor	Unit of Measure	Information Provided
Average torque	Cm	Ncm	When recorded at probing, it is a quantity measuring bone density. The mathematical relationship between Cm and density, D, (expressed as g/cm^3^), when no irrigation is used, is given by the equation D = (Cm + 11.93)/43.97 [46].
Peak torque	Cp	Ncm	When recorded at implant insertion, it is the maximum torque that was exerted by the micromotor during implant placement. In the present work, it has been indicated with the acronym IT (insertion torque).
Integral	I	Ncm^2^/100	When measured at implant insertion, it provides the area bounded by the torque–depth curve (Figure 3). If the implant threads are evenly spaced and the rotation speed at insertion is constant (two conditions that are met in this study), the integral (I) is equal to the insertion energy, IE, multiplied by a constant factor.
Depth	P	mm/10	Indicates the depth reached by the probe, when density is being measured, or by the implant when it is being placed.

**Table 3:** Parameters of the y = mx + q lines best fitting the I–density, IT–density, and RT–density plots and corresponding Pearson’s r regression coefficients. C, cylindrical; CT, cylindrical-tapered; CM, cylindrical-modified, D, density.

Plot	Implant Type	Parameters of the Regression Line		Pearson’s r
		m (slope)	q (intercept)	
I–density	C	904.4 ± 152.4	−211.3 ± 49.8	0.9027
	CT	639.8 ± 122.3	−108.1 ± 46.5	0.8795
	CM	535.2 ± 125.9	−92.4 ± 51.9	0.8325
IT–density	C	184.9 ± 38.1	−37.0 ± 12.4	0.8640
	CT	204.8 ± 23.7	−39.9 ± 9.0	0.9504
	CM	89.1 ± 27.5	+4.8 ± 11.4	0.7526
RT–density	C	255.4 ± 54.3	−60.9 ± 17.7	0.8569
	CT	190.4 ± 33.0	−47.7 ± 12.5	0.8981
	CM	90.2 ± 55.3	+3.0 ± 22.8	0.4998

I–density, IT–density, and RT–density plots for the three implant types are displayed in Figure 4.

**Table 4:** Average I, IT, and RT values for each implant types and significance p of the ANOVA tests comparing them. A significant difference among the three implants can be observed for all three implant primary stability parameters under investigation. Values reported in this table are also shown as plots in Figure 5, together with the corresponding significance of paired comparisons.

Implant Type	I	IT	RT
C	81.4 ± 48.1	22.8 ± 10.3	21.7 ± 14.3
CT	132.4 ± 40.7	37.1 ± 12.05	23.9 ± 11.8
CM	125.8 ± 42.5	41.1 ± 7.8	39.8 ± 11.9
P	0.03	0.001	0.007

**Table 5:** Statistical significance p of ANCOVA tests comparing the slopes of I–density, IT–density, and RT–density regression lines for each type of implant.

Implant Type	I vs. IT	I vs. RT	IT vs. RT
C	<0.001 *	0.001 *	0.30 (NS)
CT	0.003 *	0.003 *	0.73 (NS)
CM	0.003 *	0.005 *	0.98 (NS)

* *p* < 0.05; NS: not significant.
